# Expanded Substrate Specificity in D-Amino Acid Transaminases: A Case Study of Transaminase from *Blastococcus saxobsidens*

**DOI:** 10.3390/ijms242216194

**Published:** 2023-11-10

**Authors:** Sofia A. Shilova, Ilya O. Matyuta, Elizaveta S. Petrova, Alena Y. Nikolaeva, Tatiana V. Rakitina, Mikhail E. Minyaev, Konstantin M. Boyko, Vladimir O. Popov, Ekaterina Yu. Bezsudnova

**Affiliations:** 1 Bach Institute of Biochemistry, Research Centre of Biotechnology, Russian Academy of Sciences, Moscow 119071, Russia; zavyalovasonya@yandex.ru (S.A.S.); i.matyuta@fbras.ru (I.O.M.); lispetrova@gmail.com (E.S.P.); aishome@mail.ru (A.Y.N.); taniarakitina@yahoo.com (T.V.R.); kmb@inbi.ras.ru (K.M.B.); vpopov@inbi.ras.ru (V.O.P.); 2 Department of Chemistry, Lomonosov Moscow State University, Moscow 119991, Russia; 3 Complex of NBICS Technologies, National Research Center “Kurchatov Institute”, Moscow 123182, Russia; 4 Shemyakin & Ovchinnikov Institute of Bioorganic Chemistry, Russian Academy of Sciences, Moscow 117997, Russia; 5 N. D. Zelinsky Institute of Organic Chemistry, Russian Academy of Sciences, Moscow 119334, Russia; mminyaev@ioc.ac.ru; 6 Department of Biology, Lomonosov Moscow State University, Moscow 119991, Russia

**Keywords:** transaminase, D-amino acid, structure-function relationships, stereoselective amination, phenylhydrazine

## Abstract

Enzymes with expanded substrate specificity are good starting points for the design of biocatalysts for target reactions. However, the structural basis of the expanded substrate specificity is still elusive, especially in the superfamily of pyridoxal-5′-phosphate-dependent transaminases, which are characterized by a conserved organization of both the active site and functional dimer. Here, we analyze the structure–function relationships in a non-canonical D-amino acid transaminase from *Blastococcus saxobsidens*, which is active towards D-amino acids and primary (*R*)-amines. A detailed study of the enzyme includes a kinetic analysis of its substrate scope and a structural analysis of the holoenzyme and its complex with phenylhydrazine—a reversible inhibitor and analogue of (*R*)-1-phenylethylamine—a benchmark substrate of (*R*)-selective amine transaminases. We suggest that the features of the active site of transaminase from *B. saxobsidens*, such as the flexibility of the R34 and R96 residues, the lack of bulky residues in the β-turn at the entrance to the active site, and the short O-pocket loop, facilitate the binding of substrates with and without α-carboxylate groups. The proposed structural determinants of the expanded substrate specificity can be used for the design of transaminases for the stereoselective amination of keto compounds.

## 1. Introduction

**Scheme 1:**
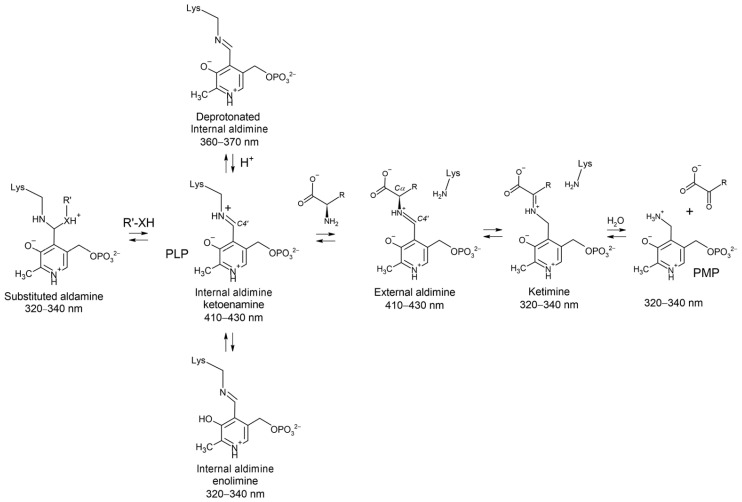
The general mechanism of the first half-reaction of the transamination catalytic cycle with indicated absorption maxima of the intermediate compounds.

Substrate specificity, catalytic efficiency, and stability are key characteristics of enzymes, determining their function in cells and repertoire of biocatalytic transformations [1,2]. The same reactions can be processed by homologous enzymes of expanded and narrow substrate specificity [3,4]. The breadth of substrate scope results from the different evolution histories and is based on the peculiarities of the active site architecture [4,5]. The superfamily of pyridoxal-5′-phosphate (PLP)-dependent transaminases (TAs) involves enzymes catalyzing the stereoselective reversible transfer of amino groups from amino acids to keto acids following a two-step Ping-Pong Bi-Bi mechanism; see Scheme 1 [6,7,8]. In cells, TAs exclusively convert α-amino acids into the respective α-keto acids and vice versa ([9,10,11]).

Being highly enantioselective, TAs can be of expanded and narrow substrate specificity [7,11]. Although the α-carboxylate group is an important recognition moiety in substrate binding, some TAs catalyze the processing of ω-amino acids, primary amines, and their prochiral keto analogues [7,11,12]. The expanded substrate specificity makes these TAs attractive candidates for the industrial stereoselective amination of various compounds [2,13,14] and a good starting point for the design of biocatalysts with new substrate specificity [15,16,17].

TAs belong to folds I and IV of PLP-dependent enzymes [18]. The catalytic unit of a TA is a homodimer with two active sites at the dimer interface, containing PLP covalently bound to the catalytic lysine [19,20,21,22]. Within each fold, the TAs are characterized by the strong similarity of the dimer structure and the architecture of the two active sites located in the dimeric interface, while different substrate specificity is achieved by the variability in the amino acid compositions of the active site [11,23]. The superfamily of TAs of PLP fold type IV include branched-chain L-amino acid TAs (BCATs, EC 2.6.1.46) converting L-amino acids and α-keto acids, D-amino acid TAs (DAATs, EC 2.6.1.21) converting D-amino acids and α-keto acids, and (*R*)-selective amine TAs converting primary (*R*)-amines and ketones ((*R*)-ATAs, EC 2.6.1.B21) [11,24]. Interestingly, some BCATs and DAATs demonstrate expanded substrate specificity: they convert primary (*R*)-amines, but they cannot convert ketones into the corresponding (*R*)-amines as true (*R*)-ATAs [25,26,27,28]. At the same time, true (*R*)-ATAs can utilize D-alanine as the donor of an amino group in the amination of ketones [29,30]; however, α-keto acids are poor substrates for (*R*)-ATAs, and the production of D-amino acids is possible only if the side chain of the substrate is small and the coproduct removal is maintained [31].

The structural basis of the expanded substrate specificity in TAs is not easy to define. A detailed study of the organization of the active site in BCATs from *Thermobaculum terrenum* [32] and *Haliangium ochraceum* [27], which were found to be active towards L-amino acids and (*R*)-1-phenylethylamine ((*R*)-PEA), showed extra hydrophobic patches and a lack of arginine residues conservative for canonical BCATs in the active sites. Moreover, the rearrangement of some hydrogen bonds made the active site more flexible and appropriate for the accommodation of (*R*)-PEA without an α-carboxylate group [27,32]. In 2016, the first TA active towards both D-amino acids and aromatic amines ((*R*)-PEA and (*R*)-1-aminotetralin) from *Curtobacterium pusillum* (CpuTA) was described [25]. Similarly to BCATs with expanded substrate specificity, CpuTA did not catalyze the transamination between ketones and amino acids. Notably, CpuTA differed from the canonical DAAT from *Bacillus sp.* (bsDAAT) [21,33,34] in the amino acid composition of the active site: no “carboxylate trap” formed by triad of residues was observed; instead, two positively charged residues—arginine and lysine—were suggested to coordinate substrates with α-carboxylate groups. The structural basis of the expanded substrate specificity and productive (*R*)-amine binding in the CpuTA remains unclear.

Recently, we discovered three TAs that contained two arginine residues in the active site, similarly to CpuTA. The novel TAs were characterized structurally and functionally. TAs from *Haliscomenobacter hydrossis* [35] and *Aminobacterium colombiense* [36,37] were found to be strictly specific towards D-amino acids and their α-keto analogues. However, TA from *Blastococcus saxobsidens* converted both D-amino acids and primary aromatic amines ((*R*)-PEA and (*R*)-1-aminotetralin). Herein, we report the detailed characterization of the TA from *B. saxobsidens* (BlasaTA). We determined the enzyme’s substrate scope and optimal reaction conditions for the conversion of D-amino acids and (*R*)-amines. We obtained the crystal structures of the holoenzyme and its complex with phenylhydrazine—an analogue of (*R*)-PEA. This allows us to suggest structural factors for the expanded substrate specificity in DAATs.

## 2. Results

### 2.1. Identification, Expression, and Purification of BlasaTA

Searching for new TAs revealed a sequence of TA of PLP fold type IV in the genome of Gram-positive bacterium *B. saxobsidens* (GenBank ID: WP_014378188.1). The new TA shared sequence identity with bsDAAT, CpuTA, Halhy, and AmicoTA of 24%, 44%, 26%, and 24%, respectively. The alignment of the sequence motifs forming the enzyme’s active site (specificity-determining motifs) showed the non-canonical organization of the active site of BlasaTA (Table 1).

The recombinant form of BlasaTA was expressed in a soluble form and purified to homogeneity (Figure S1). The molecular weight of the purified enzyme determined by gel filtration was approximately 60 kDa, indicating the homodimeric state of the enzyme (Figure S2).

### 2.2. Spectroscopic Features of BlasaTA

**Figure 1:**
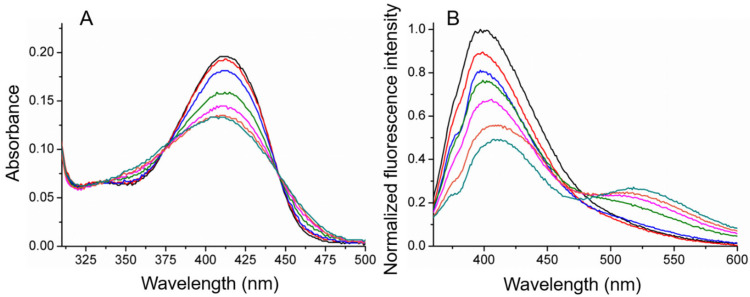
Absorption and fluorescence spectra of BlasaTA. (**A**) Absorption spectra and (**B**) fluorescence emission spectra (excitation at 340 nm) of 25 µM BlasaTA in mixed buffer 25 mM K-phosphate and 25 mM CHES at different pH: black—pH 7.0, red—pH 7.5, blue—pH 8.0, green—pH 8.6, magenta—pH 9.0, orange—pH 9.5, dark cyan—pH 10.0, at 25 °C.

The absorption spectra of BlasaTA in the PLP form at pH 7.0–10.0 exhibited a maximum at 408 nm with a small shoulder at 330 nm (Figure 1A). The maximum at 408 nm was attributed to the ketoenamine form of the internal aldimine, and the shoulder at 330 nm corresponded to several forms: the enolimine form of the internal aldimine and the substituted aldamine and deprotonated form of the internal aldimine (Scheme 1) [38]. To detail the protonation state of BlasaTA in solution, a fluorescence analysis was performed (Figure 1B). The fluorescence spectra showed an emission maximum at 400–410 nm with a shoulder at 500–525 nm when exciting at 340 nm. These spectra corresponded to both the enolimine and substituted aldamine, and the maximum at 525 nm corresponded to the ketoenamine, a byproduct of enolimine excitation [38,39]. The fluorescence maximum of the deprotonated form of the internal aldimine at 430 nm [40] was not observed in the whole pH range. Therefore, the recombinant BlasaTA exists in a protonated form in solution at pH 7.0–10.0.

### 2.3. Substrate Scope and Analysis of the Overall Transamination Reaction

The amino donor scope of BlasaTA was determined by examining the deamination of various amino compounds in a half-reaction assay (Table S1). BlasaTA was active towards various D-α-amino acids and some primary aromatic (*R*)-amines: (*R*)-1-phenylethylamine ((*R*)-PEA), (*R*)-1-(4-bromophenyl)ethylamine, (*R*)-1-(4-chlorophenyl)ethylamine, (*R*)-aminotetraline.

**Scheme 2:**
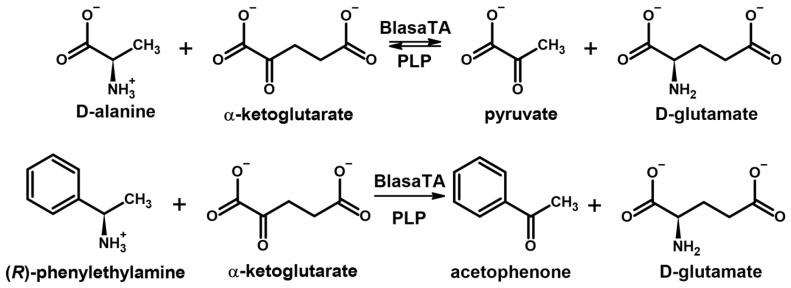
Transamination reactions, catalyzed by BlasaTA.

**Figure 2:**
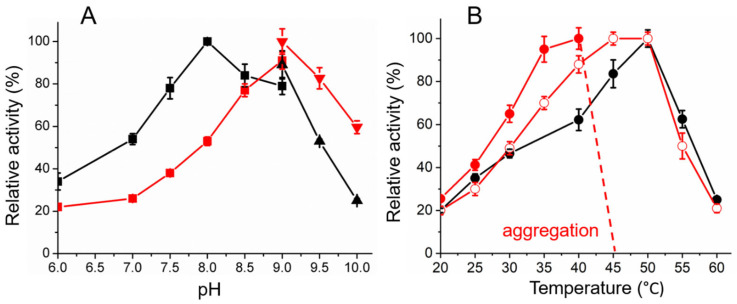
(**A**) pH and temperature dependences of activity of BlasaTA in the overall transamination reactions *D-alanine + α-ketoglutarate* (black) and *(R)-PEA + α-ketoglutarate* (red). Maximal enzyme activity measured was set as 100% relative activity. (**A**) 100% corresponds to 7.6 ± 0.1 U/mg (black), 0.043 ± 0.002 U/mg (red) at 30 °C. Black/red squares: mixed buffer 25 mM Tris-HCl and 25 mM K-phosphate, pH 6.0–9.0. Black/red triangles: 50 mM CHES, pH 9.0–10.0. (**B**) 100% corresponds to 14 ± 1 U/mg (black), 0.033 ± 0.001 U/mg (solid red circles) in 50 mM K-phosphate buffer, pH 8.0 and 0.075 ± 0.005 U/mg (open red circles) in 50 mM CHES buffer, pH 9.0. Bars denote standard deviation.

The pH and temperature dependences of the enzyme’s activity were determined in the overall transamination reactions *D-alanine + α-ketoglutarate* and (*R*)*-PEA + α-ketoglutarate* (Scheme 2, Figure 2A,B). In the reaction mixture with (*R*)-PEA and α-ketoglutarate, we observed the aggregation of BlasaTA in 50 mM K-phosphate buffer, pH 7.0–8.0, at 30–40 °C, while BlasaTA stayed active and soluble for at least one hour in the reaction mixture with *(R)*-PEA in 50 mM CHES buffer, pH 9.0, at 30–50 °C. Thus, a kinetic analysis with D-amino acids and (*R*)-amines was performed in 50 mM K-phosphate buffer, pH 8.0, 40 °C, and in 50 mM CHES buffer, pH 9.0, 30 °C, correspondingly.

Steady-state kinetic measurements of the overall transamination reactions were performed (Table 2). The specificity constants (*k_cat_/K_m_*) for D-glutamate and α-ketoglutarate were the highest among the studied substrates. The rate and efficiency of the reaction with 4-methyl-2-oxovalerate and (*R*)-PEA were significantly lower. No substrate inhibition by α-ketoglutarate was observed in a concentration range of 0–12 mM.

We did not observe the amination of ketones by BlasaTA with either D-amino acids or (*R*)-PEA as amino donors. The enantioselectivity of BlasaTA was assessed in the synthesis of D-leucine and D-phenylalanine from keto analogues, using D-glutamate as the amino donor in 100 mM K-phosphate buffer, pH 7.5 at 30 °C. A one-pot three-enzyme system was employed to shift the equilibrium of the transamination reaction towards the products. After 60 h, the product yield reached 99.2% and 62.4% for leucine and phenylalanine, respectively. The enantiomeric excess of D-leucine and D-phenylalanine reached 99.3% and 99.1%, respectively (Figure S3).

### 2.4. Analysis of the Affinity of BlasaTA for Various Substrates Using Half-Reaction Assay

To detail the affinity of BlasaTA for substrates with and without α-carboxylate groups, we carried out a half-reaction assay with glycine, D-valine, (*R*)-PEA, (*R*)-phenylpropylamine, and phenylhydrazine, a reversible inhibitor and structural analogue of (*R*)-PEA (Table 3). The rate of deamination and the specificity of BlasaTA for amines increased from pH 8.0 to 9.0, accompanied by an increase in the value of KD, thus supporting not only the reactivity of the amines in the deprotonated form but the reorganization of the active site as well. If changes in pH only affected the concentration of the deprotonated amine, the value of kmax would remain constant over the entire pH range.

The reaction between the PLP form of BlasaTA and phenylhydrazine was limited to the formation of the external aldimine (transimination step), whose accumulation was observed at 370 nm. Notably, the pKa of the amino group of phenylhydrazine is significantly lower than that of other aromatic substrates (Table 3). The kinetic parameters remained constant over the pH range. This could be due to the following: (1) phenylhydrazine reacts in a deprotonated form at both pH 8.0 and 9.0, and (2) the reorganization of the active site does not affect the transimination step [41,42].

The specificity constant of BlasaTA for substrates with α-carboxylate groups changed to a lesser extent from pH 8.0 to 9.0. An increase in the kmax value indicated the reorganization of the active site with changes in pH. From the obtained results, the assistance of the α-carboxylate group in the deprotonation (activation) of the substrates in the transimination step [36] is not obvious. However, the similarity of the KD values of the examined substrates confirms the adjustment of the O-pocket for the binding of the α-carboxylate groups of D-amino acids and the aromatic moieties of amines.

**Table 3:** Kinetic parameters of half-reactions between the PLP form of BlasaTA and aromatic amino donors at 30 °C.

Amino Donor	Structure	50 mM K-Phosphate Buffer, pH 8.0			50 mM CHES Buffer, pH 9.0			pKa of the Amino Group
		k_max_, s^−1^	K_D_, mM	k_max_/K_D_, M^−1^ s^−1^	k_max_, s^−1^	K_D_, mM	k_max_/K_D_, M^−1^ s^−1^	
Glycine	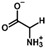	0.072 ± 0.004	7 ± 1	10 ± 2	0.14 ± 0.01	4.5 ± 0.6	31 ± 6	9.78 [41]
D-Valine	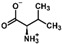	0.22 ± 0.01	204 ± 7	1.07 ± 0.06	0.69 ± 0.06	360 ± 60	1.9 ± 0.4	9.74 [41]
D-Phenylalanine	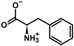	0.171 ± 0.005	32 ± 2	5.3 ± 0.5	0.71 ± 0.03	67 ± 5	11 ± 1	9.09 [43]
(*R*)-1-Phenylethyl amine	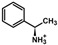	0.0044 ± 0.0001	19 ± 2	0.23 ± 0.03	0.43 ± 0.02	180 ± 20	2.3 ± 0.3	9.41[44]
(*R*)-1-Phenylpropylamine	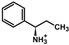	0.000243 ± 0.000004	6.1 ± 0.3	0.040 ± 0.008	-	>45 *	0.20 ± 0.01	9.56 **
Phenylhydrazine	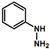	1.19 ± 0.01	31 ± 1	38 ± 2	0.77 ± 0.03	47 ± 5	16 ± 2	5.21 [45]

*—protein precipitation is observed at 50 mM (*R*)-1-phenylpropylamine. **—pKa is predicted using Playground server: https://playground.calculators.cxn.io/ (accessed on 1 September 2023).

### 2.5. Organization of the Functional Dimer of BlasaTA

**Figure 3:**
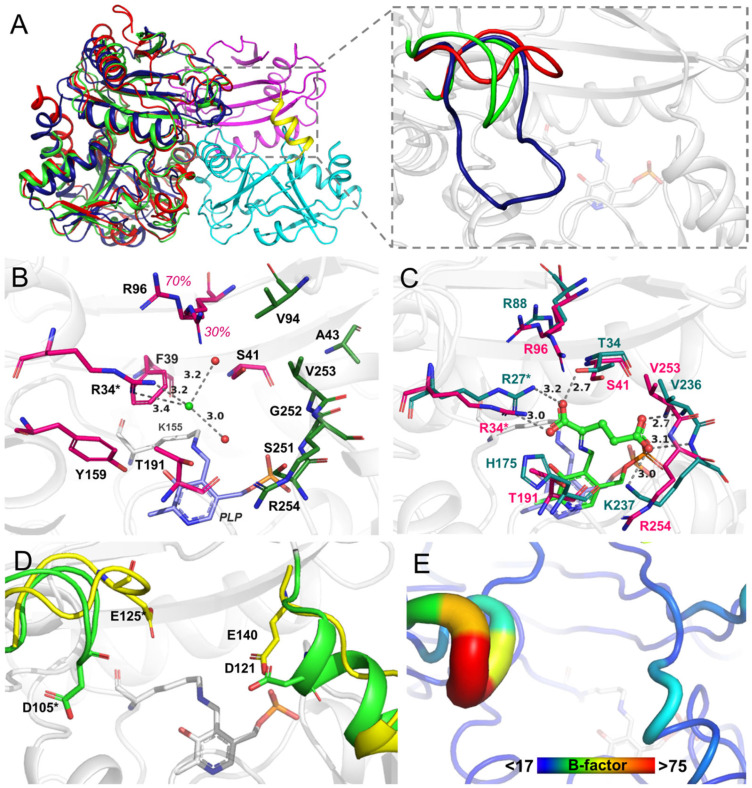
Crystal structure of BlasaTA. (**A**) Function dimer of BlasaTA. In the right subunit, the small domain is colored in magenta, the large domain in cyan, and the interdomain loop in yellow. The left subunit of BlasaTA (green) is superposed with the subunits of CpuTA (PDB ID: 5K3W, red) and AmicoTA (PDB ID: 8AHR, dark blue). Insert: A magnified view of the O-pocket loop (the other part of the molecules are shown transparently). (**B**) Active site of BlasaTA (the O-pocket residues are colored in magenta, the P-pocket residues in green, the PLP molecule in lilac, the chloride ion in light green, water molecules in red). Two conformations of R96 are shown with corresponding occupancies indicated. (**C**) The superposition of the active site of BlasaTA and the active site of AmicoTA in complex with D-glutamate (PDB ID: 8AYK) (residues of BlasaTA are shown in magenta, those of AmicoTA in dark cyan, the PLP molecule in BlasaTA is colored in lilac, the ketimine of PLP and D-glutamate in light green). (**D**) The superposition of the O-pocket loops and the interdomain loops of BlasaTA (light green) and CpuTA (yellow). (**E**) B-factor putty representation of the entrance to the active site of BlasaTA (orientation is similar to panel D). (*) indicates residues from the adjacent subunit.

The crystal structures of the PLP form of BlasaTA (PDB ID 8PNW) and the complex with phenylhydrazine (PDB ID 8PNY) were determined at 1.7 and 1.8 Å resolution, respectively (Table S2). Crystal contact analysis confirmed a dimeric state both for the holoenzyme and the complex. The RMSD for the Cα atoms between the subunits of the holoenzyme and the complex did not exceed 0.2 Å, indicating no gross structural changes upon substrate binding. The organization of the functional dimer of BlasaTA is typical of PLP fold type IV TAs [20,21,22,23] (Figure 3A) (Table S3). One subunit comprises two α/β domains: a small domain (residues 1–117) and a large domain (residues 130–281), connected by an interdomain loop (residues 118–129). The O-pocket loop of BlasaTA (residues 100–109) is short, compared to the ones observed in the functional dimers of TAs of PLP fold type IV (13–17 residues [20,21,22,23,46,47]), except CpuTA, whose O-pocket loop is also short (Table S4) (Figure 3A). The functional dimer of BlasaTA contains two symmetric active sites, formed by amino acid residues of both subunits. In the holoenzyme, the electron density of the PLP molecule is well defined, clearly revealing the covalent bond with the catalytic K155. In the complex, the electron density in the active site corresponds to the mixture of the adduct of PLP and phenylhydrazine, mimicking the external aldimine (Scheme 1), and an apoform of BlasaTA with equal occupancies. The phosphate group of the cofactor is bound in a manner typical of TAs of PLP fold type IV (Figure S4). Besides the covalent link to K155, the pyridine ring of PLP forms a hydrogen bond with Y159 and E188 and is additionally sandwiched between the side chain of L212 and backbone atoms of T191 (β-turn).

### 2.6. Organization of the Active Site of Holoenzyme and Complex with Phenylhydrazine

Both the O-pocket and P-pocket of the active site of BlasaTA are lined by hydrophobic residues with the inclusion of positively charged guanidine groups of the arginine residues (Figure 3B). The residues R34*, F39, S41, and R96 are conserved among the non-canonical DAATs (Table 1 and Table S4) [25,35,36,37]. Unlike R88 in AmicoTA [36,37] and R90 in Halhy [35], R96 in BlasaTA does not form a hydrogen bond network with the surrounding residues and is present in two conformations in the active site (similarly to K117 in CpuTA) (Figure 3B). The residue R34* does not form any hydrogen bonds either. Next, no bulky residues are observed at the active site entrance in BlasaTA, contrary to the active site organization in AmicoTA and Halhy (Table S4). The chloride ion from the crystallization buffer is found in the O-pocket of the holoenzyme near R34* (Figure 3B). When superposing the structures of the holoenzyme of BlasaTA and the complex with D-glutamate of AmicoTA (PDB ID 8AYK), the chloride ion accommodates in the O-pocket, similarly to the α-carboxylate group of D-glutamate in the complex; this binding site in BlasaTA is formed by residues R34*, S41, R96, and T191 (Figure 3C). According to the superposition, the residues of the β-turn ^251^SGVR^254^ are involved in the coordination of the γ-carboxylate group of D-glutamate (Figure 3C). The O-pocket and interdomain loops contain negatively charged residues D105* and D121, similar to E125* and E121 in CpuTA, and form an electrostatic surface charge at the entrance to the active site (Figure 3D). The B-factors of the residues of the O-pocket loop in BlasaTA are higher than those of other residues, thus indicating the mobility of the loop and the flexibility of its residues (Figure 3E).

**Figure 4:**
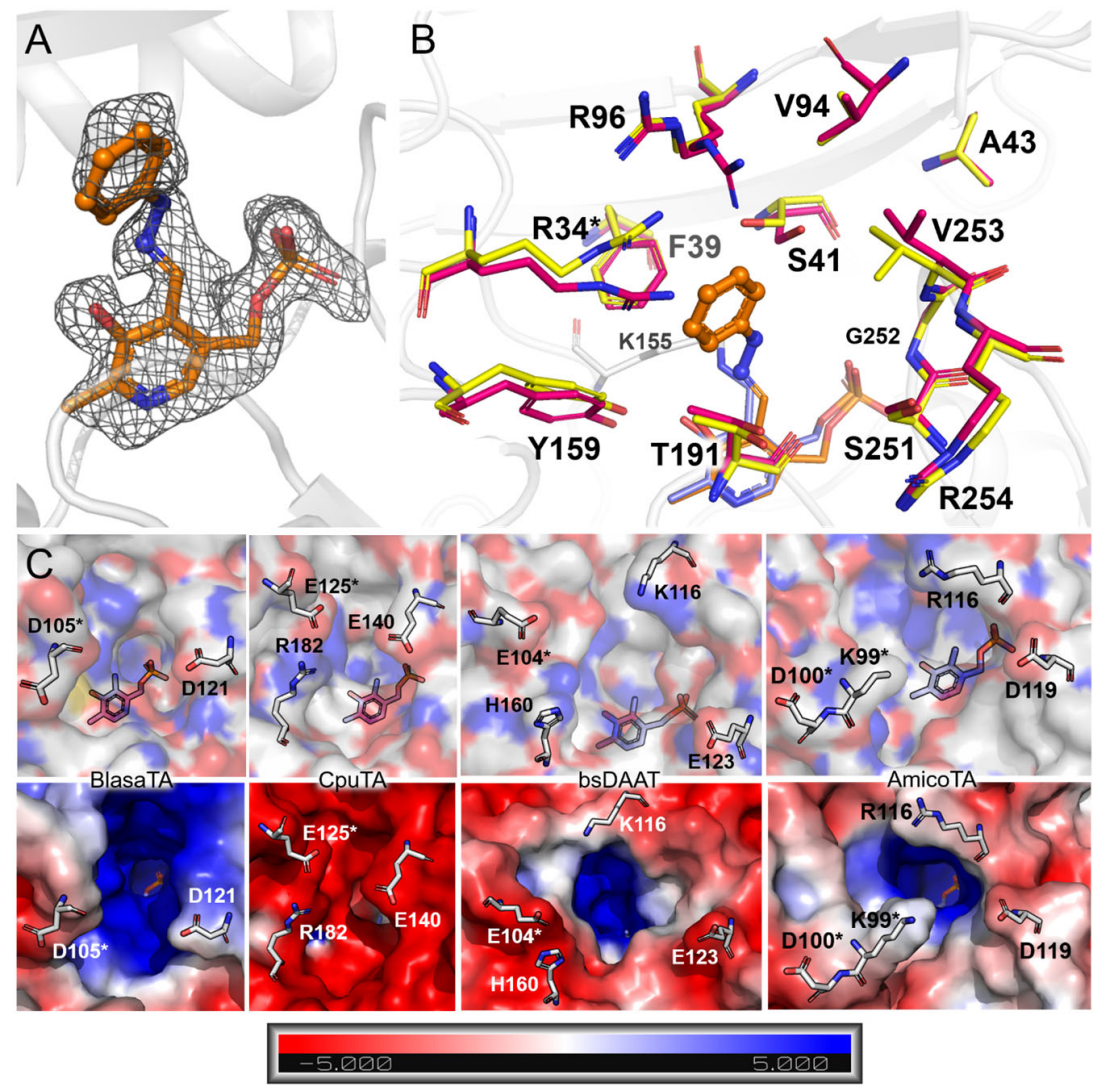
Analysis of the active site of BlasaTA. (**A**) Polder map of the adduct of PLP with phenylhydrazine depicted at 3σ level. (**B**) The superposition of the active sites of the holoenzyme and the complex with phenylhydrazine (residues of the holoenzyme are colored in magenta, those of the complex in yellow). (**C**) Surface (top) and electrostatic surface at pH 9.0 (down) representation of the entrances to the active sites of DAATs. (*) indicates residues from the adjacent subunit. The PLP molecule is shown in lilac, the adduct of phenylhydrazine and PLP in orange.

In the structure of the complex with phenylhydrazine, the linkage between phenylhydrazine and PLP is clearly observed in the electron density map (Figure 4A), with a dihedral angle C3-C4-C4α-N of about 40°. The pyridine ring of the cofactor is tilted by 12° around the N1-C6 bond from its position in the holoenzyme. The released ε-NH_2_ group of K155 forms a hydrogen bond with the N4′ atom of the adduct. Notably, the accommodation of the phenyl ring in the O-pocket is accompanied by the formation of stacking interactions with the side chain of F39 [48] and the removal of the side chains of residues R96 and R34* from the active site (Figure 4B). In the apoenzyme and holoenzyme, the residues R96 and R34* are oriented inside the active site (Figure 3B). The orientation of the D105* and D121 residues is slightly changed, resulting in the shortening of the distance between the oxygen atoms of γ-carboxylate groups from 12.3 Å in the holoenzyme to 11.00 Å in the complex.

## 3. Discussion

The effective enzymatic transamination includes the proper binding of a substrate in the active site and an available proton transfer system for substrate activation [8,9,10,42] (according to the mechanism of enzymatic transamination, the substrate (amine or amino acid) should be deprotonated when the Michaelis complex is forming). Extended substrate specificity means that both of these conditions are met for substrates of different natures. BlasaTA is active towards D-amino acids, α-keto acids, and primary aromatic (*R*)-amines at pH 8.0–9.0: the activity towards D-amino acids is significantly higher than that towards (*R*)-amines. Considering the protonation states of both the internal aldimine of BlasaTA and the amino groups of substrates (D-amino acids and (*R*)-amines), the assistance of the α-carboxylate group in the deprotonation of D-amino acids may be a marked advantage when the Michaelis complex is forming [36]. Recently, a proton transfer system exploring the conserved histidine residue was suggested for (*R*)-ATAs [49]; however, no similar system was observed in BlasaTA or its homologue, CpuTA. At the same time, CpuTA is characterized by similar values of activity towards D-amino acids and primary aromatic (*R*)-amines [25]. For BlasaTA, the rate of deamination of amines is higher at pH 9.0, when the deprotonated form becomes significant.

BlasaTA and CpuTA, together with AmicoTA and Halhy, are non-canonical DAATs, distinguished by specificity-determining arginine or lysine residues in the active site. In the O-pocket of BlasaTA, the α-carboxylate group of the substrate appears to be coordinated by the conserved R34* and R96, accompanied by S41 and T19. The binding of D-glutamate and α-ketoglutarate can be strengthened by the coordination of the γ-carboxylate group in the P-pocket. Despite the similarity in the active site organization, several structural features of BlasaTA and CpuTA lead to extended substrate specificity, whereas AmicoTA and Halhy are active exclusively towards D-amino acids. The flexibility of R96 and R34* (K117 and R51* in CpuTA) allows the binding of both the α-carboxylate group and the phenyl moiety in the O-pocket of the active site (in Halhy and AmicoTA, the similar arginine residues are fixed by a hydrogen bond network). The short O-pocket loop and the lack of a bulky residue at the entrance to the active site in BlasaTA and CpuTA (contrary to H175 in AmicoTA or R179 in Halhy) make the active site more accessible for bulky substrates. Notably, no switch in open/closed conformations was observed for these O-pocket loops, whereas this is observed in (*R*)-ATAs [28,46]. Previously, two negatively charged glutamate residues at the entrance to the active site of CpuTA were described as a feature [25]. We found these pairs of negatively charged residues at the entrance of canonical and non-canonical DAATs (Figure 4C). Whether these pairs can assist in the deprotonation of substrates via accepting a proton is unclear: in BlasaTA and CpuTA, these residues are distanced at 11.0–12.3 Å and 6.6 Å, respectively; in bsDAAT and AmicoTA, they are at 15.6 Å and 13.9 Å, respectively. Considering the mobility of the O-pocket loop, one can expect the approaching of the residues with the formation of the “proton trap”; however, we did not observe this approaching in the crystal structures of the complexes (PDB ID: 8AYK, 8PNY, 8AYJ). Notably, the active site entrance surface is charged differently in BlasaTA and CpuTA (Figure 4C), with BlasaTA being similar to (*R*)-ATAs from Gram-negative bacteria *Shinella* ([28], PDB ID: 6XU3) and CpuTA for “true” (fungal) (*R*)-ATAs.

To summarize, the flexible arginine in the active site is observed in PLP fold type I aromatic TAs, ornithine TAs, etc., whereas a variable volume due to the mobility of the O-pocket loop, bearing a flexible arginine residue for pyruvate coordination, is observed in (*R*)-ATAs of PLP fold type IV. Thus, these structural features in TAs can be considered effective for the binding of substrates without α-carboxylate groups, with a distant carboxylate group, and with α-carboxylate groups in the same active site.

## 4. Materials and Methods

### 4.1. Cloning, Expression, and Purification of the Recombinant BlasaTA

Gene BlasaTA_4517 encoding PLP type IV transaminase (282 a.a., 29.1 kDa) was identified in the complete genome sequence of *B. saxobsidens* (strain DD2) [50]. The optimized nucleotide sequence (http://genomes.urv.es/OPTIMIZER/ (accessed on 1 February of 2022)) was synthesized with 5′ and 3′ restriction sites NdeI and HindIII and then cloned into the pET-21d vector (Novagen, Darmstadt, Germany), modified as described in [51] to create a protein with a (His)_6_-tag and a TEV protease site at the N-terminus. The *Escherichia coli* Rosetta (DE3) pLysS cells (Novagen, Germany) containing the expression vector were grown in LB medium with 100 μg/mL ampicillin and 34 μg/mL chloramphenicol (Panreac-AppliChem, Darmstadt, Germany) at 37 °C until an OD_600_ of 0.8. Protein expression was induced by 0.2 mM IPTG for 18 h at 24 °C. The cells were collected by centrifugation, resuspended in 50 mM K-phosphate buffer, pH 8.0, supplemented with 500 mM NaCl, 20 mM imidazole, 2 M urea, 5 mM β-mercaptoethanol, 10% (*v*/*v*) glycerol, 0.2 μg/mL lysozyme, 100 μM PLP, and 1 mM PMSF, and disrupted by sonication. The crude cell extract was treated with 0.1 mg DNAse (Sigma-Aldrich, St Louis, MO, USA) and centrifuged for 45 min at 18,500× *g* at 4 °C. The supernatant was filtered through a 0.45 μm filter (Millipore, Burlington, MA, USA) and loaded onto a 5 mL HisTrap HP column (Cytiva, Marlborough, MA, USA) equilibrated with 50 mM K-phosphate buffer, pH 8.0, containing 500 mM NaCl, 20 mM imidazole, and 0.1% (*v/v*) Triton X-100. (His)_6_-tagged recombinant BlasaTA was eluted by a linear gradient from 20 to 500 mM imidazole in the same buffer without Triton X-100. The target protein was incubated with 1 mM PLP for 1 h at 25 °C, concentrated to 20–25 mg/mL using a 30 kDa centrifuge filter (Millipore, Burlington, MA, USA), and transferred to a storage buffer (50 mM K-phosphate, pH 8.0, containing 100 mM NaCl, 1 mM β-mercaptoethanol, and 100 μM PLP). The protein was stored at −20 °C with 50% glycerol. For biochemical characterization, (His)_6_-tagged BlasaTA was used.

For crystallization, the (His)_6_-tagged recombinant BlasaTA was mixed with (His)_6_-tagged TEV protease (1 mg per 10 mg of the transaminase) and incubated overnight at 4 °C in 50 mM HEPES buffer, pH 7.5, containing 10% (*v/v*) glycerol, 1 mM EDTA, 5 mM β-mercaptoethanol, and 100 μM PLP. The solution was then dialyzed against 50 mM K-phosphate buffer, pH 8.0, containing 500 mM NaCl, 20 mM imidazole, 5 mM β-mercaptoethanol, and 20 μM PLP, and applied to a HisTrap HP column (Cytiva, Marlborough, MA, USA). The BlasaTA without (His)_6_-tag was collected in the flow-through mode, concentrated, and further purified on a Superdex 200 10/300 GL column (Cytiva, Marlborough, MA, USA) equilibrated in 50 mM HEPES buffer, pH 8.0, containing 100 mM NaCl, 100 μM PLP, and 1 mM DTT. The resulting fraction of recombinant BlasaTA was concentrated to 15–20 mg/mL and frozen at −70 °C. The (His)_6_-tag cleavage efficiency and the protein purity were analyzed by SDS-PAGE (12%). The protein concentrations were determined spectrophotometrically at 280 nm using the calculated extinction coefficient (https://web.expasy.org/protparam/ (accessed on 1 June 2022)). The amino acid sequences were checked by MALDI-TOF MS analysis (UltraFlextreme Bruker Daltonik, Bremen, Germany).

### 4.2. Half-Transamination Reaction Assay

The PLP form of BlasaTA was obtained by incubating BlasaTA with an excess of PLP and α-ketoglutarate for 1 h at 25 °C, followed by transfer into 50 mM K-phosphate buffer, pH 8.0, or 50 mM CHES buffer, pH 9.0, using a 5 mL desalting column (Cytiva, Marlborough, MA, USA). Half-transamination reactions between the PLP form of BlasaTA (30–35 μM) and different amino compounds (0–250 mM) were monitored spectrophotometrically at 408 nm by measuring the decrease in the aldimine concentration in 50 mM K-phosphate buffer, pH 8.0, or 50 mM CHES buffer, pH 9.0, at 30 °C in microtiter plates (UV-Star, Greiner Bio-One GmbH, Frickenhausen, Germany) using a SPECTROstar Omega plate reader (BMG Labtech, GmbH, Ortenberg, Germany). The rate constants of the half-reactions were determined by fitting Equation (1):

(1) $$A_{t}=A_{\infty }+∆Aexp(-k_{obs}t)$$

where $A_{t}$ is the absorbance at time t, ΔA is the difference between the absorbance at t = 0 and t = ∞, $A_{\infty }$ is the final absorbance, and k_obs_ is the observed rate constant. The maximal rate constant k_max_, the dissociation constant K_D_, and the specificity constant k_max_/K_D_ were obtained by fitting Equation (2):

(2) $$k_{obs}=\frac{k_{max}[S]}{K_{D}+[S]}$$

All measurements were performed at least in triplicate, and the data were analyzed using the Origin 8.0 software (OriginLab, Northampton, MS, USA).

### 4.3. Enzyme Activity Assay

The activity of BlasaTA in transamination reactions with D-alanine and D-glutamate as amino donors was assessed spectrophotometrically using the second enzymatic reaction with lactate dehydrogenase from rabbit muscle (Roche Diagnostic GmbH, Mannheim, Germany) (LDH assay) or (*R*)-2-hydroxyglutarate dehydrogenase (HGDH assay), respectively, in the presence of NADH. The recombinant HGDH from *Acidaminococcus fermenats* was obtained as described in [36]. The reaction progress was monitored by detecting a decrease in absorbance at 340 nm (ε(NADH) = 6.22 mM^−1^ cm ^−1^) in microtiter plates (UV-Star, Grenier, Germany) using a SPECTROstar Omega plate reader (BMG Labtech, Ortenberg, Germany). The activity of BlasaTA with (*R*)-PEA as the amino donor was determined using a direct photometric assay by detecting acetophenone formation (acetophenone-assay) at 245 nm (ε(acetophenone) = 11.6 mM^−1^ cm ^−1^), using an Evolution 300 UV–Vis spectrophotometer (Thermo Scientific, Waltham, MA, USA). After a preincubation period of 5 min, the reaction was initiated by the amino donor. The activity of BlasaTA was calculated from the initial linear region of the reaction’s progress curve. One unit (U) was defined as the amount of the enzyme that catalyzed the conversion of 1 μmol of substrate into a product per minute.

### 4.4. Effects of pH and Temperature on the Overall Transamination Reaction

pH and temperature effects were analyzed in the overall transamination reactions between 2 mM α-ketoglutarate and 5 mM D-alanine or 5 mM (*R*)-PEA. The optimal pH was determined at 30 °C using mixed buffer of 25 mM Tris-HCl and 25 mM K-phosphate, pH 6.0–9.0, and 50 mM CHES buffer, pH 9.0–10.0. The effect of temperature on the reaction rate was studied in 50 mM K-phosphate buffer, pH 8.0.

### 4.5. Determination of Kinetic Parameters of the Overall Transamination Reactions

The kinetic parameters of the overall reactions catalyzed by BlasaTA were determining using the Michaelis–Menten model. The reactions with D-alanine were analyzed by the LDH assay, those with D-glutamate by the HGDH assay, and those with (*R*)-PEA by the acetophenone assay. The reactions with D-alanine and D-glutamate were studied in 50 mM K-phosphate buffer, pH 8.0, containing 0.04–0.08 μM of the purified BlasaTA, 30 μM PLP, 330 μM NADH, and 3 U/mL LDH or HGDH, respectively, at 40 °C. The reactions with (*R*)-PEA were studied in 50 mM CHES buffer, pH 9.0, containing 1–2 μM of the purified BlasaTA and 30 μM PLP, at 30 °C. The substrate concentrations for the overall reactions varied in the following ranges: 0.05–25 mM D-alanine, 0.05–12 mM D-glutamate, 2.5–20 mM (*R*)-PEA, 0.1–12 mM α-ketoglutarate, 1–100 mM 4-methyl-2-oxovalerate, and 0.5–25 mM pyruvate. The substrate saturation curves were generated at constant co-substrate concentrations. The kinetic parameters were calculated by fitting the initial velocity data to Equation (3):

(3) $$V=\frac{V_{max}\times A\times B}{K_{m}^{A}\times B+K_{m}^{B}\times B+A\times B}$$

where *V* is the initial velocity, *V_m_* is the maximal velocity, *A* and *B* are the substrate concentrations, and $K_{m}^{A}$ and $K_{m}^{B}$ are the *K_m_* of substrates A and B, respectively. All measurements were performed at least in triplicate. The data were analyzed using the Origin 8.0 software.

### 4.6. Analysis of the Product Yield and Enantiomeric Excess in the Transamination Reaction

Product yields were determined in reactions catalyzed by BlasaTA *4-methyl-2-oxovalerate* + *D-glutamate* and *phenylpyruvate* + *D-glutamate* by measuring the consumption of 4-methyl-2-oxovalerate or the formation of D-phenylalanine. To shift the equilibrium towards the products, a one-pot three-enzyme system was employed. The coproduct, α-ketoglutarate, was removed from the reaction mixture using the HGDG assay while recovering NADH in D-glucose conversion, which was catalyzed by glucose dehydrogenase. The reaction mixture contained 100 mM K-phosphate buffer, pH 7.5, 200 μM PLP, 100 mM D-glutamate, 50 mM 4-methyl-2-oxovalerate or 50 mM phenylpyruvate, 2 mg/mL BlasaTA, 1 mM NADH, 150 mM D-glucose, 180 U/mL HGDH, and 30 U/mL glucose dehydrogenase (Sigma-Aldrich, St. Louis, MO, USA). The reaction mixtures were incubated at 30 °C for 60 h. The reactions were terminated by removing the enzyme using an Amicon-Ultra-15 centrifugal tube (Millipore, USA), and then the filtrate was analyzed by HPLC (ÄKTA Purifier, Marlborough, MA, Cytiva, USA) using a reverse-phase C18 column (Zorbax Eclipse XDB-C18, 5 µm, 4.6 mm × 150 mm (Agilent, Santa Clara, CA, USA)). The chiral analysis of the produced D-leucine and D-phenylalanine was performed by HPLC using a reverse-phase C18 column with a UV detector set at 340 nm. D-amino acid products were modified. The HPLC and derivatization conditions are described in the Supplementary Materials.

### 4.7. Crystallization and Data Collection

The BlasaTA was crystallized by the “hanging drop” vapor diffusion method in 24-well VDX plates (Hampton Research, Aliso Viejo, CA USA). An amount of 1.5 µL of protein (15 mg/mL) was mixed with 1.5 µL of precipitant containing 0.1 M HEPES, pH 7.5, 3.7–4.1 M NaCl and set up over 500 µL of precipitant in a sealed reservoir at 277 K. To obtain crystals of the complex of BlasaTA with phenylhydrazine, the holoenzyme crystal was soaked in a crystallization solution containing 20 mM of the ligand for 2 min.

Datasets for the holoenzyme and complex with phenylhydrazine were collected at 100 K at the ID23-1 beamline (ESRF, Grenoble, France) [50] and Rigaku OD XtaLAB Synergy-S (IOC RAS, Moscow, Russia), accordingly. The former dataset was indexed, integrated, and scaled using the Dials, Version 3.17.0 [52] program from the software package CCP4, Version 1.7.009 [53], while the processing of the latter one was performed with the CrysAlisPro software v.1.0.43 (Oxford Diffraction/Agilent Technologies UK Ltd., Yarnton, UK). In both cases, the space group was suggested by Pointless, Version 1.12.15 [54] as P3_1_21 (Table S2).

### 4.8. Structure Solution and Refinement

The structure of the holo form of BlasaTA was solved by the molecular replacement method using the MOLREP program, Version 11.0 [55] with the atomic coordinates of aminotransferase from *Mycobacterium tuberculosis* (PDB ID: 6Q1R) as a starting model, while the structure of the BlasaTA complex was solved using the BlasaTA holo form structure. In both structures, one copy of the protein was found in an asymmetric unit. The refinement of all structures was carried out using the REFMAC5 program of the CCP4 suite, Version 5.7.0009 [53] (Table S2). The visual inspection of the electron density maps and the manual rebuilding of the model were carried out using the COOT interactive graphics program, Version 0.9.8.91 [56]. The isotropic B-factor and the hydrogen atoms in fixed positions were used during the refinement.

### 4.9. Structure Analysis and Validation

The visual inspection of the modeled structure was carried out using the COOT program v.1 [56] and the PyMOL Molecular Graphics System, Version 4.6 (Schrödinger, New York, NY, USA). The structure comparison and superposition were performed using the PDBeFOLD program, Version 2.58 [57]. The dimer contacts were analyzed using the PDBePISA program, Version 1.48 [58]. The surface charge was calculated with the APBS electrostatic plugin for PyMOL, Version 3.4.1 [59].

## 5. Conclusions

We characterized a new transaminase from the Gram-positive bacterium *B. saxobsidens* (BlasaTA) with expanded substrate specificity. The enzyme is active towards various D-amino acids and primary aromatic (*R*)-amines at pH 8.0–9.0. According to the analysis of the crystal structures of the holoenzyme and the complex with phenylhydrazine, an analogue of the substrate (*R*)-1-phenylethylamine, the active site of BlasaTA, shares common features with another non-canonical DAAT of expanded substrate specificity from *C. pusillum*. Both DAATs are distinguished by flexible arginine residues in the active site and the adjustable volume of the active site cavity. The findings deepen our understanding of the structural basis of the expanded and promiscuous substrate specificity in transaminases and are useful for the design of biocatalysts with target specificity.

## Figures and Tables

**Table 1:** The sequence motifs in the active site of canonical (bsDAAT), non-canonical DAATs [25,34,35,36], and BlasaTA.

DAAT	Amino Acids Forming the Active Site
bsDAAT	^26^FxxxxYxVxK^35^ … ^88^YxQ^90^ … ^98^RxH^100^ … ^178^GSSS^181^ … ^240^STTS^243^
AmicoTA	^27^RxxxxFxTxS^36^ … ^88^RxY^90^ … ^99^KxH^101^ … ^173^GSHS^176^ … ^234^GTVK^237^
Halhy	^28^RxxxxFxYxL^37^ … ^90^RxV^92^ … ^99^xxx^101^ … ^177^SARS^180^ … ^238^STIK^243^
CpuTA	^46^RxxxxFxTxA^60^ … ^117^KxI^119^ … ^126^xxx^128^ … ^208^GPTS^211^ … ^270^SSVR^273^
BlasaTA	^34^RxxxxFxSxA^43^ … ^96^RxF^98^ … ^104^xxx^106^ … ^189^GPTS^192^ … ^251^SGVR^254^

**Table 2:** Kinetic parameters of the overall transamination reactions, catalyzed by BlasaTA.

Substrate	Co-Substrate	*k_cat_*, s^−1^	*K_m_*, mM	*k_cat_/K_m_*, M^−1^ s^−1^
**50 mM K-phosphate buffer, pH 8.0, 40 °C**				
D-alanine	5 mM α-ketoglutarate	8.1 ± 0.3	2.0 ± 0.3	4000 ± 800
α-ketoglutarate	25 mM D-alanine	8.1 ± 0.3	0.53 ± 0.06	15,300 ± 2300
D-alanine	100 mM 4-methyl-2-oxovalerate	0.190 ± 0.004	0.14 ± 0.01	1360 ± 130
4-methyl-2-oxovalerate	25 mM D-alanine	0.190 ± 0.004	45 ± 2	4.2 ± 0.3
D-glutamate	15 mM pyruvate	7.5 ± 0.3	0.40 ± 0.06	18,800 ± 3600
pyruvate	15 mM D-glutamate	7.5 ± 0.3	1.6 ± 0.1	4700 ± 500
D-glutamate	100 mM 4-methyl-2-oxovalerate	0.27 ± 0.01	0.19 ± 0.01	1400 ± 130
4-methyl-2-oxovalerate	5 mM D-glutamate	0.27 ± 0.01	56 ± 3	3.5 ± 0.3
**50 mM CHES buffer, pH 9.0, 30** **°** **C**				
(*R*)-PEA	2 mM α-ketoglutarate	0.053 ± 0.002	6.6 ± 0.4	8.0 ± 0.8
α-ketoglutarate	20 mM (*R*)-PEA	0.053 ± 0.002	0.025 ± 0.003	2100 ± 300
(*R*)-PEA	2 mM pyruvate	0.074 ± 0.003	8.0 ± 0.7	9 ± 1
pyruvate	20 mM (*R*)-PEA	0.074 ± 0.003	0.027 ± 0.003	2700 ± 400
(*R*)-PEA	10 mM 4-methyl-2-oxovalerate	0.032 ± 0.001	4.5 ± 0.4	7.1 ± 0.9
4-methyl-2-oxovalerate	20 mM (*R*)-PEA	0.032 ± 0.001	2.0 ± 0.2	16 ± 2

## Data Availability

The data presented in this study are available on request from the corresponding author.

## Supplementary Materials

The following supporting information can be downloaded at: https://www.mdpi.com/article/10.3390/ijms242216194/s1.
