# Supplementary material for: Expanded Substrate Specificity in D-Amino Acid Transaminases: A Case Study of Transaminase from Blastococcus saxobsidens
Source: Int J Mol Sci. 2023 Nov 10;24(22):16194. doi: 10.3390/ijms242216194 (PMC10671532; doi:10.3390/ijms242216194)
Supplement: Supplementary file 1 [file ijms-24-16194-s001.zip › ijms-2701396-supplementary.pdf]

Supplementary

International Journal of Molecular Sciences

Expanded substrate specificity in D-amino acid transaminases: a case study of transaminase from *Blastococcus saxobsidens*

Sofia A. Shilova [1], Elizaveta S. Petrova [1,2], Ilya O. Matyuta[1], Alena Y. Nikolaeva[1,3], Tatiana V. Rakitina[1,4], Mikhail E. Minyaev[5], Konstantin M. Boyko[1], Vladimir O. Popov[1], and Ekaterina Yu. Bezsudnova [1,*]

[1]Bach Institute of Biochemistry, Research Centre of Biotechnology, Russian Academy of Sciences, Moscow, Russia;
[2]Department of Chemistry, Lomonosov Moscow State University, Moscow, Russia
[3]Complex of NBICS Technologies, National Research Center "Kurchatov Institute", Moscow, Russia;
[4]Shemyakin&Ovchinnikov Institute of Bioorganic Chemistry, Russian Academy of Sciences, Moscow, Russia
[5]N.D. Zelinsky Institute of Organic Chemistry, Russian Academy of Sciences, Moscow, Russia
[6]Department of Biology, Lomonosov Moscow State University, Moscow, Russia

*****Correspondence: eubez@inbi.ras.ru; eubez@yandex.ru

Content:

Figure S1-S2……………………………………………………………………..p. 2

Figure S3…………………………………………………………………………p. 3

Figure S4…………………………………………………………………………p. 4

Table S1………………………………………………………………………..…p. 5

Table S2………………………………………………………………………..…p. 6

Table S3………………………………………………………………………..…p. 7

Table S4………………………………………………………………………..…p. 8

Supplemented figures

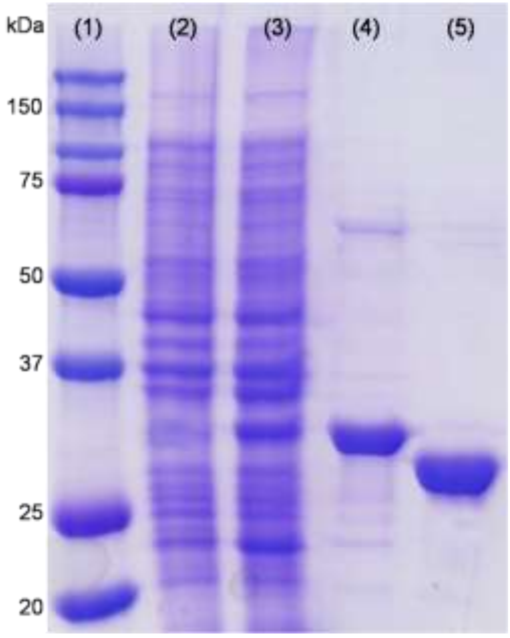

Figure S1. SDS-PAGE of steps of expression and purification of BlasaTA. (1) Precision Plus Protein Dual Color Standards (Bio-Rad, USA) (2) *E. coli* cell lysate before induction; (3) *E. coli* cell lysate after IPTG induction; (4) fraction of BlasaTA-$(His)_6$TEV-tag after HisTrap HP column; (5) fraction of BlasaTA after cleavage of $(His)_6$-tag, using TEV-protease, and following by gel-filtration.

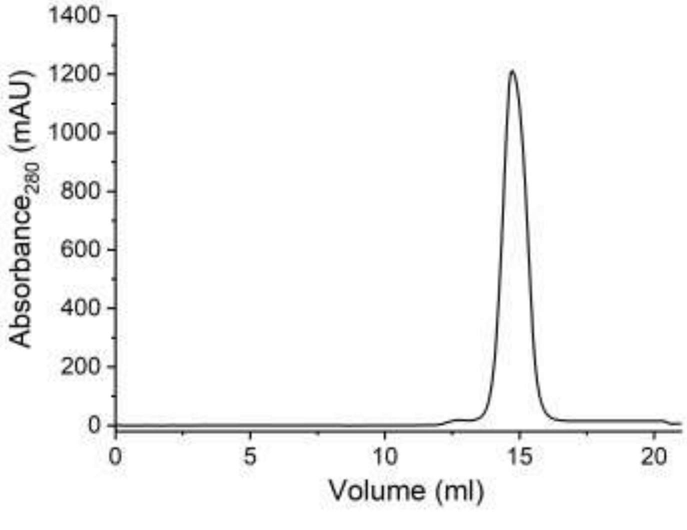

Figure S2. The gel filtration elution profile for BlasaTA. The major peak corresponds to the BlasaTA dimer. Chromatography was carried out on a 24 mL Superdex 200 10/300 GL column (Cytiva, USA) equilibrated with 50 mM HEPES buffer, pH 8.0, containing 100 mM NaCl, 1 mM DTT, and 100 μM PLP.

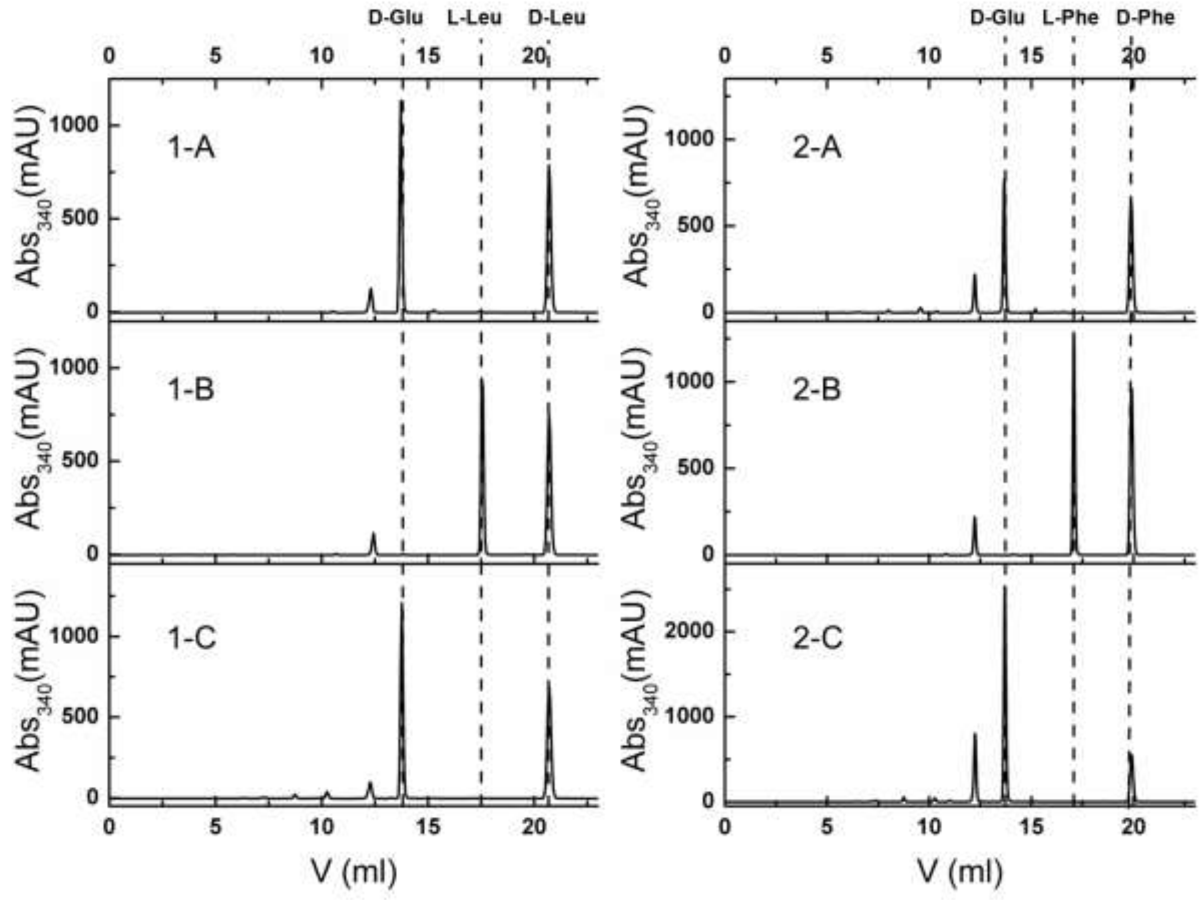

Figure S3. Determination of the enantiomeric excess in the reactions of *D-glutamate + 4-methyl-2-oxovalerate* (reaction I) and *D-glutamate + phenylpyruvate* (reaction II) catalyzed by BlasaTA. Chromatograms of standards and sample (leucine, phenylalnine and glutamate) derivatized with Marfey's reagent. 1-A: reference standards of D-glutamate and D-leucine at concentrations of 50 mM; 1-B: reference standards of D- and L-leucine at concentrations of 50 mM; 1-C: the reaction I sample. 2-A: reference standards of D-glutamate and D-phenylalanine at concentrations of 50 mM; 2-B: reference standards of D- and L-phenylalanine at concentrations of 50 mM; 2-C: the reaction II sample.

Derivatization conditions

10 μL of the reaction solution was mixed with 25 μL of 10 mM Marfey's reagent ($N_α$-(2,4-dinitro-5-fluorophenyl)-L-alanine amide, Sigma, USA) (2.5 eq) in acetonitrile and 10 μL of 1 M $NaHCO_3$ and incubated at 50 °C for 2 h. The reaction mixture was cooled to room temperature, and then the reaction was stopped by adding 3 μL of 4 M HCl and 10 μL of 100% ethanol.

HPLC analysis conditions

The yields determination (method A):

Eluent – 20 mM Na-phosphate buffer, pH 3.0, 15% methanol
Flow rate – 1.0 mL/min
Injection volume – 20 μL

Detection – UV, 210 nm

The chiral analysis (method B):

Eluent A – 0.1% trifluoroacetic acid in water
Eluent B – 0.1% trifluoroacetic acid in methanol
Flow rate – 1.0 mL/min
Gradient – 0-15 min: 20-70% B
Injection volume – 10 μL
Detection – UV, 340 nm

Retention parameters

**Compound**	**V_{ret}, mL**		**t_{ret}, min**	
	Method A			
4-Methyl-2-oxovalerate	7.6		7.6	
D-Phenylpyruvate	4.3		4.3	
	Method B			
	L-isomer	D-isomer	L-isomer	D-isomer
Leucine*	17.6	21.1	17.6	21.1
Phenylalanine*	17.0	19.9	17.0	19.9
Glutamate*	-	14.3	-	14.3

*After derivatization with Marfey's reagent

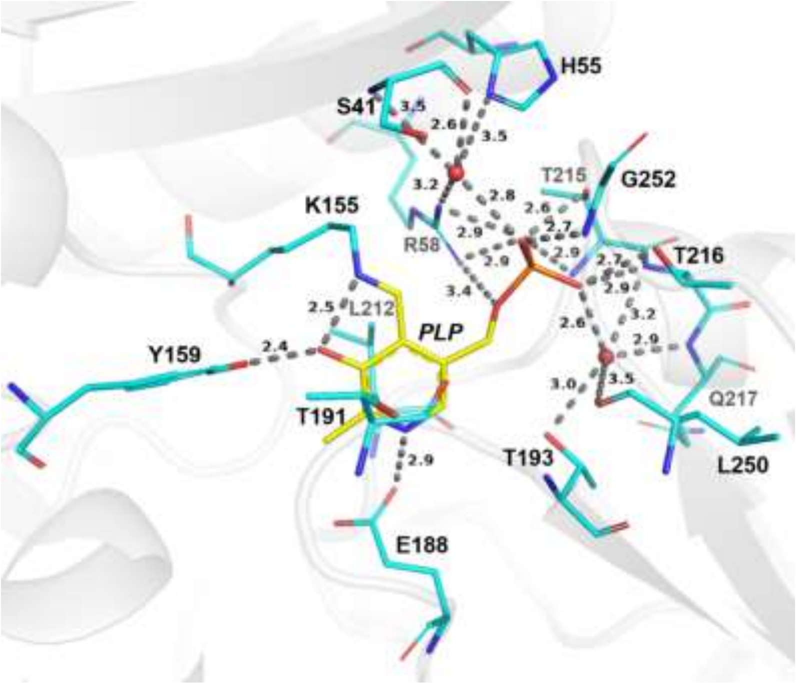

Figure S4. Binding of PLP molecule in the BlasaTA. PLP molecule is shown in yellow, water molecules are shown as red balls, distances are given in angstroms (Å) and shown as dashed lines.

Supplemented tables

Table S1. The observed rate constants of half-reactions between the PLP-form of BlasaTA (35 μM) and amino donors in concentration of 5 mM in 50 mM K-phosphate buffer, pH 8.0, at 30 °C. ND- not detected.

Amino acid	Amino acid structure	k_{obs}, s^{-1}	Amine	Amine structure	k_{obs}, s^{-1}
D-Glutamate*	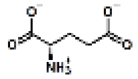	1.3 ± 0.1	(*R*)-1-Phenylethylamine	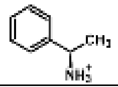	0.00162 ± 0.00006
D-Alanine*	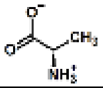	0.9 ± 0.1	(*R*)-1-(4-Chlorophenyl)ethylamine	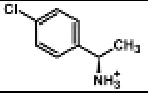	0.0105 ± 0.0001
D-Aspartate*	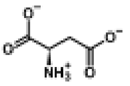	0.43 ± 0.05	(*R*)-1-(4-Bromophenyl)ethylamine	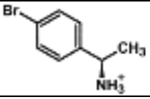	0.0055 ± 0.0003
D-Serine	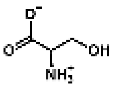	0.13 ± 0.01	(*R*)-1-Phenylpropylamine	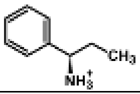	0.000144 ± 0.000005
D-Valine	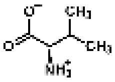	0.00091 ± 0.00001	(*R*)-1-Aminotetraline	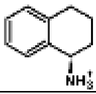	0.00836 ± 0.00005
D-Norvaline	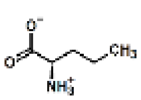	0.75 ± 0.08	(*R*)-1-(1-Naphthyl)ethylamine	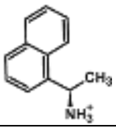	ND
D-Leucine	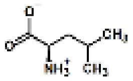	0.051 ± 0.006	Benzhydrylamine	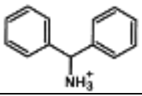	ND
D-Phenylalanine	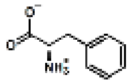	0.018 ± 0.003	(*R,S*)-2-Amino-5-methylhexane	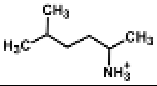	ND
D-Tryptophane	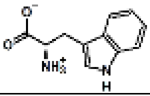	0.52 ± 0.05	(*R,S*)-1,3-Dimethylbutylamine	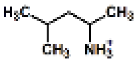	ND
D-Ornithine	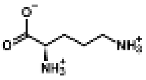	0.005 ± 0.001	(*S*)-1-Phenylethylamine	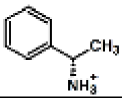	ND
D-Lysine	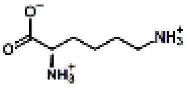	0.0018 ± 0.0001	(*S*)-1-Aminotetraline	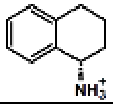	ND
L-Glutamate	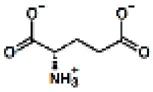	ND	(*R,S*)-1-Methyl-3-phenylpropylamine	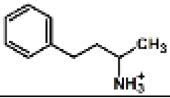	ND
L-Alanine	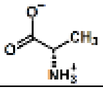	ND	3-Phenyl-1-propylamine	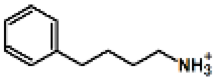	ND
L-Leucine	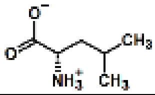	ND	Isobutylamine	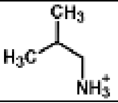	ND
D,L-β-Aminobutanoic acid	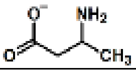	ND	Isopropylamine	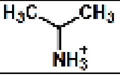	ND
γ-Aminobutanoic acid	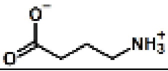	ND	D,L-Alaninol	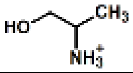	ND

*half-reactions were performed with 100 μM of amino donor

Table S2. Data collection, processing and refinement.

	**BlasaTA holo form**	**BlasaTA complexed with phenylhydrazine**
**Diffraction source**	ESRF (ID23-1 beamline)	Rigaku OD XtaLAB Synergy-S
**Wavelength (Å)**	0.97	1.54
**Temperature (K)**	100	
**Detector**	PILATUS 6M	HyPix-6000HE
**Crystal-to-detector distance (mm)**	125.5	31.5
**Rotation range per image (°)**	0.1	0.25
**Total rotation range (°)**	120	240
**Space group**	$P3_121$	$P3_121$
***a, b, c* (Å)**	105.94, 105.94, 51.32	104.64, 104.64, 51.54
**α, β, γ (°)**	90.0, 90.0, 120.0	90.0, 90.0, 120.0
**Average mosaicity (°)**	0	1.19
**Resolution range (Å)**	52.97-1.70 (1.73-1.70)	22.59-1.80 (1.84-1.80)
**Completeness (%)**	97.4 (91.5)	99.6 (96.6)
**Average redundancy**	3.5 (2.8)	12.9 (12.8)
$\langle I/\sigma(I)\rangle$	10.1 (1.3)	10.7 (1.9)
**Rmeas (%)**	9.5 (61.3)	22.6 (158.2)
$CC_{1/2}$	99.4 (79.2)	99.6 (50.6)
R_{fact} **(%)**	17.1	20.3
$R_{free.}$ **(%)**	19.2	25.4
**RMSD Bonds (Å)**	0.01	0.01
**RMSD Angles (°)**	1.69	1.79
**Most favored (%)**	97.8	96.0
**Allowed (%)**	2.2	4.0
**PDB entry code**	8PNW	8PNY

Table S3. Superpositions of the BlasaTA subunit with subunits of PLP fold types IV TAs.

TA from (PDB ID)	Activity type	RMSD, Å	Sequence identity, %
Geoglobus acetivarans (5E25)	BCAT	1.6	26
Curtobacterium pusillum (5K3W)	DAAT + R-TA	1.7	40
Haliscomenobacter hydrossis (7P7X)	DAAT	1.7	24
Aminobacterium colombiense (8AHR)		1.7	19
Bacillus sp. YM-1 (4DAA)		1.7	20
Aspergillus fumigatus (4UUG)	R-TA	1.8	19
Nectria heamatococca (4CMD)		1.9	21
Thermobaculum terrenum (6GKR)	BCAT + R-TA	1.9	17

Table S4. Structure-based sequence alignment of BlasaTA and DAATs. Amino acid composition of secondary structural elements forming the active sites of DAATs. Crystal structures of the following DAATs were analyzed: DAAT from *Bacillus* sp. YM-1(PDB ID 1DAA), from *B. sphaericus* (4TM5), from *C. pusillum* (5K3W), from *H. hydrossis* (7P7X) and from *A. colombiense* (8AHR). Amino acid residues known to participate in substrate binding are shown in red. Similar amino acid residues in homologs are shown in Bold. Positively charged residues in the P-pockets are shown in Bold and *Italic*. Canonical DAAT are highlighted in rose, non-canonical DAATs are shown in green.

DAAT from	O-pocket α-helix	βX-strand	βY-strand	O-pocket loop	Interdomain loop	β-turn1	β-turn2
Bacillus sp. YM-1	^{21}DRGYG**F**G^{27}	^{29}GV**Y**EVV***K***VY^{37}	^{85}GHI**Y**FQVT^{92}	^{93}RGTSP**RAH**QFPENTVKP^{109}	^{117}NPRPLENLEKG^{128}	^{178}GSSS^{181}	^{240}STTS^{243}
B. sphaericus	^{22}DRGYQ**F**G^{28}	^{30}GI**Y**EVI***K***VY^{38}	^{86}GHV**Y**FQIT^{93}	^{94}RGTTS**R**N**H**IFPDASVPA^{110}	^{119}GERSIEQFEKG^{129}	^{179}CSSA^{182}	^{241}SVSS^{244}
A. colombiense	^{22}DLIIQ**R**G^{28}	^{30}GV**F**E**TIS**TH^{38}	^{85}TMV**R**PYIT^{92}	^{93}GDSFGKDHLFSSSRYFV^{110}	^{115}IRKPDPILYEKG^{126}	^{173}GS**H**S^{176}	^{234}GTV***K***^{237}
H. hydrossis	^{23}DLSIL**R**G^{29}	^{31}GI**F**D**YFL**AR^{39}	^{87}AGI**R**LVLT^{94}	^{95}GGYSPDGYTVNP^{107}	^{115}DLPASAWEFSAQG^{127}	^{177}SA**R**S^{180}	^{238}STI***K***^{241}
C. pusillum	^{46}DLGIT**R**G^{52}	^{54}GV**F**E**TIA**VI^{62}	^{114}LFA**K**LILT^{121}	^{122}RGIEGEGRP^{130}	^{139}GEDFSQQRLG^{148}	^{208}GP**TS**^{211}	^{270}SSV***R***^{273}
B. saxobsidens	^{29}DLGLG**R**^{34}	^{37}GI**F**E**S**VAV^{44}	^{93}GVC**R**LFLT^{100}	^{101}RGLGDGTPP^{109}	^{118}VPADTLRQRAEG^{129}	^{189}GP**TS**^{192}	^{251}SGV***R***^{254}
